# A Concise Review of Biomolecule Visualization

**DOI:** 10.3390/cimb46020084

**Published:** 2024-02-02

**Authors:** Hui Li, Xinru Wei

**Affiliations:** College of Information Science and Technology, Beijing University of Chemical Technology, Beijing 100029, China; 2020210440@mail.buct.edu.cn

**Keywords:** molecular visualization, computer graphics, representation models, level of detail, augmented reality

## Abstract

The structural characteristics of biomolecules are a major focus in the field of structural biology. Molecular visualization plays a crucial role in displaying structural information in an intuitive manner, aiding in the understanding of molecular properties. This paper provides a comprehensive overview of core concepts, key techniques, and tools in molecular visualization. Additionally, it presents the latest research findings to uncover emerging trends and highlights the challenges and potential directions for the development of the field.

## 1. Introduction

Visualization plays a crucial role in the study of biomolecules. It uses computer graphics techniques to present complex 2D or 3D molecular structures in a more intuitive and interactive way. This helps researchers understand key information, such as atomic spatial arrangements and the connectivity of chemical bonds. As a result, they can gain valuable insights into the relationship between the properties of these molecules and their structures.

Visualization technology has evolved in parallel with the advancement of structural biology. Initially, physical models made of materials like wood and brass were used for 3D molecular visualization [1,2]. With the progress in computer technology, researchers started combining computer graphics with molecular structure visualization, leading to the development of molecular graphics [3]. The continuous improvement of computer hardware has enhanced the visual representation of molecular models [4], prompting researchers to invest in molecular visualization software [5]. Additionally, animation techniques have been employed to depict molecular motion [6]. Using 3D printing technology, one can construct physical models of molecules using atomic coordinates. It represents the spatial relationships of three-dimensional molecules in a more realistic way and has gradually become a beneficial tool for teaching and the exchange of scientific research [7].

The advancements in visualization technology and tools have greatly contributed to new discoveries in structural biology. In the 1990s, the popularity of internet technology fostered the development of web-based molecular visualization tools [8]. GPU-accelerated graphics generation and rendering, as well as large-scale parallel computing, have played a crucial role in improving real-time interactive visualization effects, including geometric primitive rendering, occlusion culling, and lighting models [9].

In recent years, the increasing complexity and scale of molecular data have facilitated the development of multi-scale visualization techniques [10]. The introduction of head-mounted displays has revolutionized interactive visualization modes and driven the growth of immersive visualization [11]. Creative aesthetics and design methods have provided more effective and visually appealing representations of molecular visualization [12].

**Figure 1:**
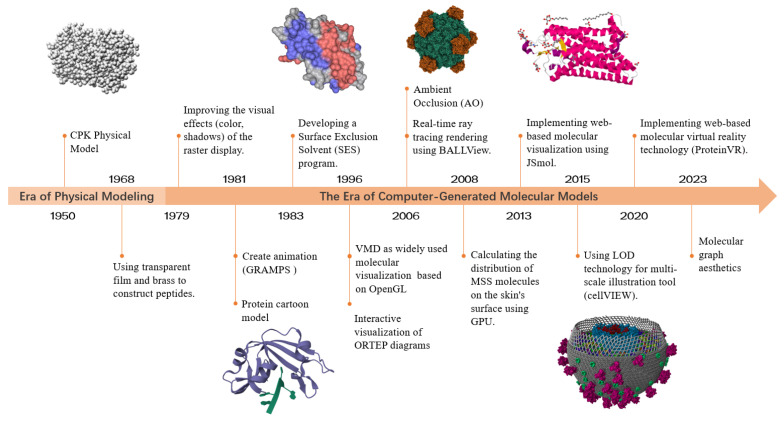
The development of molecular visualization. Specically, the figure contains various molecular graphics: the CPK model of the green fluorescent protein (PDB ID: 1EMA), the cartoon model of ribonuclease (PDB ID: 1M07), the SES surface of protein isomerase (PDB ID: 1OGZ), the AO rendering effect of viral protein (PDB ID: 1RB8), the cartoon model of visual pigment (PDB ID: 3PQR), and the medium-scale model of HIV. These graphics were generated using Materials Studio (https://www.3ds.com/products-services/biovia/products/molecular-modeling-simulation/biovia-materials-studio/ (accessed on 15 October 2023)) [13], MegaMol (https://megamol.org/ (accessed on 15 October 2023)) [14], Molstar (https://molstar.org/ (accessed on 15 October 2023)) [15], YASARA View (http://www.yasara.org/ (accessed on 15 October 2023)) [16], and the molecular viewer integrated in RCSB PDB (https://www.rcsb.org/3d-view/ (accessed on 15 October 2023)).

Figure 1 showcases the process of the development of molecular visualization, highlighting key timelines and important events. Reflecting on this history, the latest trends in development include the growing complexity of molecular data, artistic representation of molecular graphics, and the diversification of interaction methods.

There have been numerous reviews in the field of molecular visualization in recent years. Table 1 presents some recent reviews on three-dimensional molecular structure visualization. Among them, Olson [6] provides a comprehensive and specific overview of the historical development of molecular visualization. Kozlíková et al. [17] collect 241 articles and conduct a detailed review of molecular visualization techniques from various perspectives. Johnson et al. [18], in addition to their review, propose a series of methods to assist readers in selecting appropriate visualization tools and creating effective visual images based on their needs. Martinez et al. [19] further analyze and summarize advanced molecular visualization literature in the fields of structural biology and computer graphics, emphasizing the importance of cross-disciplinary integration in this field.

Despite the availability of existing reviews, this article aims to investigate the latest technologies and methods in molecular visualization due to the continuous emergence of new research achievements. This review focuses on surveying visualization technologies and tools for biomolecules, comprehensively reviewing visualization techniques for addressing specific problems, providing detailed introductions to common types of molecular visualization tools in different usage scenarios, analyzing the challenges faced by molecular visualization research, and exploring possible development trends.

The review paper is organized as follows: The review begins with a brief introduction to the historical development and recent advancements in molecular visualization. Next, it explores different representation models and their applicable scenarios. It compiles effective methods for improving the quality of three-dimensional visualizations, as well as an overview of Level of Detail techniques used to accelerate the rendering of complex molecular visualizations.

Lastly, the article summarizes the main approaches used for visualizing positional uncertainty and presents the current state of research on immersive visualization.

## 2. Visualization Technology

This section explores and summarizes representation models, rendering techniques, Level Of Detail (LOD) techniques, location uncertainty visualization techniques, and immersive visualization techniques. It presents both classic achievements and the latest progress.

### 2.1. Representation Models

**Figure 2:**
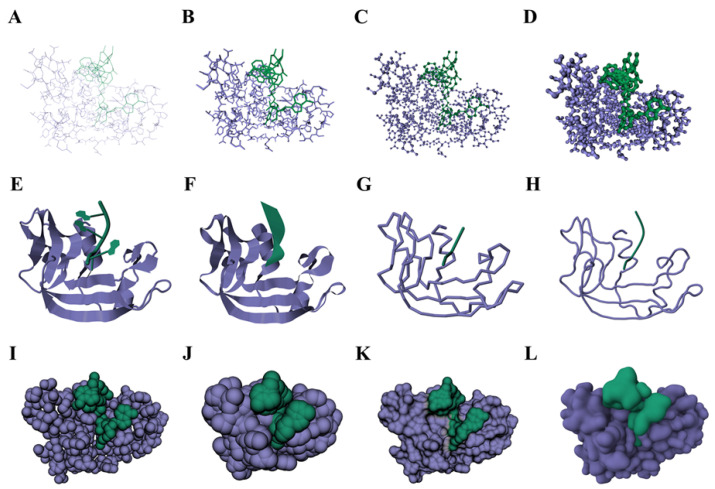
The different representation models of Ribonuclease (PDB ID: 1M07) including: (**A**) lines model, (**B**) stick model, (**C**) ball-and-stick model, (**D**) HyperBall model, (**E**) cartoon model, (**F**) ribbon model, (**G**) backbone model, (**H**) trace model, (**I**) vdW surface (space-filling model), (**J**) SAS surface, (**K**) SES surface, and (**L**) Gaussian surface. UnityMol (http://www.baaden.ibpc.fr/umol/ (accessed on 15 October 2023)) [26], MegaMol (https://megamol.org/ (accessed on 15 October 2023)) [14], and the integrated molecular viewer in RCSB PDB (https://www.rcsb.org/3d-view/ (accessed on 15 October 2023)) are used for visualization. Different colors represent separate chains within a molecule.

Biological molecules exhibit high complexity and diversity, and are composed of atoms connected through specific chemical bonds and interactions. To express their three-dimensional configuration and spatial arrangement, different representation models can be used, as shown in Figure 2. This section categorizes representation models into skeletal models, cartoon models, and surface models.

Skeletal models describe the backbone structure of molecules, including atoms, chemical bonds, and their spatial topology. They use simple geometric shapes like line segments, spheres, cylinders, and polyhedra to represent the model’s structure. Skeletal models are one of the simplest and oldest representation methods. Most molecular visualization software currently implements skeletal models, such as lines models, stick models, ball-and-stick models, and space-filling models.

The lines model (Figure 2A) is a representation method using lines to connect atoms and emphasizes the basic features and geometric relationships within the structure. The stick model (Figure 2B) is similar to the lines model, but it uses sticks to connect atoms. Ball-and-stick models (Figure 2C) and space-filling models (Figure 2I) use spheres to represent atoms, with each sphere’s radius typically being proportional to the corresponding atomic radius. This facilitates an understanding of the relative positions and structural relationships of atoms in the molecule. Space-filling models can also represent the spatial occupancy of atoms through the size and position of the spheres, revealing gaps and channels in the molecular structure. In addition to these common representation models, Chavent et al. [27] introduced a new method called HyperBall (Figure 2D), which uses hyperboloids to connect atoms represented in the form of spheres. HyperBall achieves a higher rendering efficiency and quality than triangle meshes inspired by GPU ray casting techniques.

Cartoon models integrate the representation of atoms and chemical bonds into ribbon-like or tubular structures, highlighting overall structural features. They are commonly used for proteins. Richardson [28] first proposed using ribbons and arrows to represent protein secondary structure, and Carson et al. [29] were the first to implement it programmatically. Cartoon representations of proteins are integrated into almost all molecular visualization software. Classic cartoon models (Figure 2E) and ribbon models (Figure 2F) can highlight protein secondary structure features and describe protein folding behavior. Backbone models (Figure 2G) show the folding of the polypeptide chain by creating artificial “backbone” bonds between alpha carbons.Trace models (Figure 2H) use a smooth curve to display the backbone. The hermite spline curve passes through the mid-points between alpha carbon atoms [30]. They are commonly used to emphasize the overall structure and topological relationships of proteins. Researchers have focused on improving the visualization performance of cartoon models through mesh refinement techniques and GPU acceleration techniques [17]. Recently, Borzov [31] proposed a new method using signed distance fields and sphere tracing techniques for cartoon protein representation, explaining the underlying mathematical characteristics and comparing them with existing mesh refinement methods. Ozvoldik et al. [32] successfully introduced LOD techniques into grid-based molecular model visualization, achieving cartoon model renderings for larger-scale protein data.

Surface models are obtained by computing the surfaces connecting the molecule to the surrounding environment, helping us to understand the interactions between molecules resulting from various chemical bonds. The most basic surface model is the Van der Waals Surface (vdW) (Figure 2I) [33], which is the outer surface formed by the combination of all atomic spheres in the space-filling model. A surface model shows the volume occupied by the molecule, with the radius of the atomic spheres being proportional to the Van der Waals radius. Lee and Richards created the Solvent Accessible Surface (SAS) (Figure 2J) [34], defined as the surface formed by the rolling motion of solvent molecule probes on the vdW surface. It displays all regions from which solvent molecules can enter the molecule but cannot accurately represent the volume of the molecule. Richards and Greer further researched and defined the Solvent Excluded Surface (SES) (Figure 2K) [33,35] as the surface formed by the probes’ contact points with the molecule, which accurately reflects the molecular volume and expresses the accessibility of the molecule, helping to describe the interactions between the molecule and its surrounding environment.

Blinn [36] proposed the Gaussian convolution surface (Figure 2L) implicit surface modeling algorithm, commonly used in electron density analysis to simulate the electron density map of molecular structures. From a mathematical perspective, the SES surface is the first smooth molecular surface and has attracted extensive attention and research. Connolly [37] developed a program to calculate and display the SES surface. Chavent et al. [38] used a GPU to compute a completely continuous molecular skin surface, pioneering the application of GPU in molecular surface calculation. Recent research in this field focuses on fast construction algorithms for SES surfaces, utilizing high-performance GPU acceleration or general-purpose CPU acceleration without hardware limitations. Examples include the GPU algorithms by Hermosilla et al. [39], Martinez et al. [40], Schäfer et al. [41], Alhazzazi et al. [42], and the CPU algorithm by Rau et al. [43].

Martinez et al. [40] completed an open-source implementation of the work carried out by Hermosilla et al. [39] and developed the QuickSES library to integrate it into molecular viewers, providing a standalone program that reads PDB files and outputs a complete SES mesh as a Wavefront OBJ file. In addition, new methods for constructing molecular surfaces have emerged. Hermosilla [44] introduced the use of transparency to improve the visual perception of molecular surfaces and proposed a fast method for calculating and implementing rendering of transparent and translucent materials. Bruckner [45] proposed a dynamic visibility-driven molecular surface visualization method based on Gaussian models, allowing for the dynamic high-quality surface visualization of molecules composed of millions of atoms. Wei et al. [46] introduced a machine learning algorithm that can predict classical SES surfaces on proteins and complex structures, achieving a higher computational efficiency and over 95% accuracy compared to CPUs.

### 2.2. Rendering Technology

**Figure 3:**
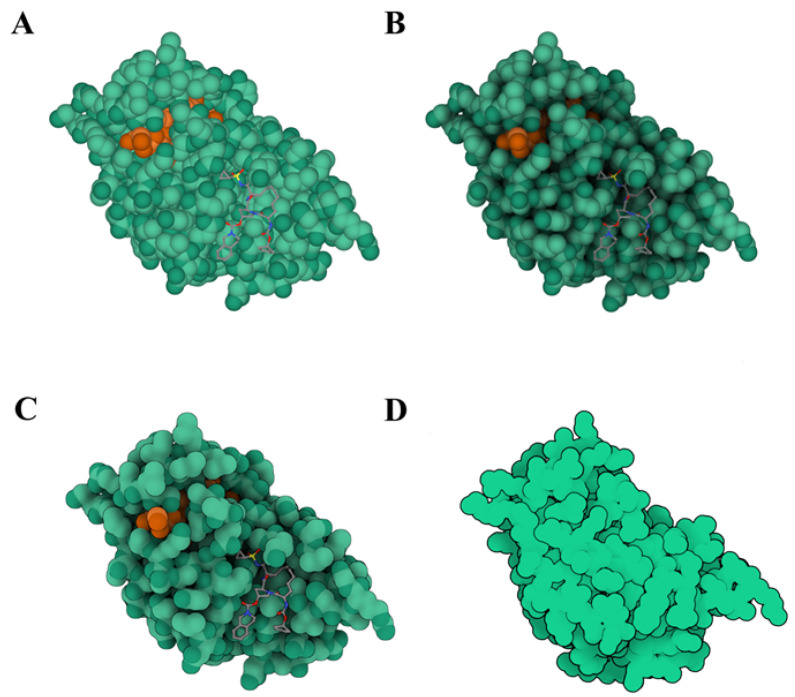
The protease inhibitor (PDB ID: 4KTC) can be rendered in four different ways: (**A**) local illumination rendering, (**B**) ambient occlusion rendering, (**C**) illustrative rendering, and (**D**) PDB-101 style illustration. Molstar [15] and Illustrate [47] are used for visualization. Different colors represent separate chains within a molecule.

The field of molecular visualization utilizes various rendering techniques to enhance the visual representation of molecules. These techniques include ray tracing, ambient occlusion, illustrative rendering, non-photorealistic rendering, and color mapping. Figure 3 showcases the various rendering effects of cartoon models and molecular surfaces.

Initially, macromolecule shadow surfaces were created using rasterization rendering [48]. However, ray tracing techniques have gradually gained popularity in molecular visualization. BALLView [49], one of the earliest molecular visualization tools, combines real-time ray tracing rendering, which has become a focus of research into visualization rendering methods. Achieving real-time performance in rendering requires algorithm parallelism and designing acceleration structures. Stone [50] summarizes interactive ray tracing and compatible rendering techniques applicable to molecular visualization and provides example codes.

To improve rendering quality, lighting models can be used to simulate reflection, refraction, and other real-world object phenomena. The Blinn–Phong local lighting model proposed by Phong [51] and Blinn [52] significantly enhances the quality and speed of real-time rendering. However, this model cannot express shadows cast by atoms onto each other in molecular visualization. To address this, ambient occlusion (AO) techniques have been proposed by Miller [53] and Zhukov [54]. AO techniques approximate global lighting models by collecting diffuse reflection information from around objects. They capture scene details, enhance stereo perception, and improve realism. For example, Tarini et al. [55] combined ambient occlusion and edge highlighting using a GPU-accelerated algorithm to enhance the real-time visualization of molecular space-filling models. Matthews et al. [56] proposed a per-pixel ambient occlusion algorithm suitable for visualizing the dynamic scenes of proteins. Hermosilla et al. [57] proposed a universal global lighting model for various molecular models. Zerari et al. [58] combined SSAO (screen space ambient occlusion) technology with multiple importance sampling techniques to accelerate real-time rendering. Rau et al. [43] utilized CPU-based ray tracing technology to interactively visualize SES (solvent-excluded surface) surfaces, achieving high-quality rendering by combining the techniques into AOOM (Ambient Occlusion Opacity Mapping).

Illustrative rendering and non-photorealistic rendering techniques create artistic effects which are different to traditional realistic rendering, imitating hand-drawn, cartoon, sketch, and other art styles. Lawonn et al. [59] reviewed illustrative rendering techniques and their practical applications, not limiting them to molecular structure visualization. Koch et al. [60] proposed a molecular illustrative representation method utilizing screen space lighting algorithms, aiding in perceiving the hierarchical structure of multi-scale models. Liang et al. [61] presented a GPU-based boundary ellipsoid abstraction representation that emphasizes the surface details of molecules using contours, making molecular visualization visually appealing and informative. Illustrative rendering and non-photorealistic rendering simplify complexity and highlight key features, making them suitable for education and communication. The education website PDB-101 (https://pdb101.rcsb.org/ (accessed on 15 October 2023)) maintained by RCSB PDB uses 2D illustrations of molecular models generated directly by Illustrate [47].

**Figure 4:**
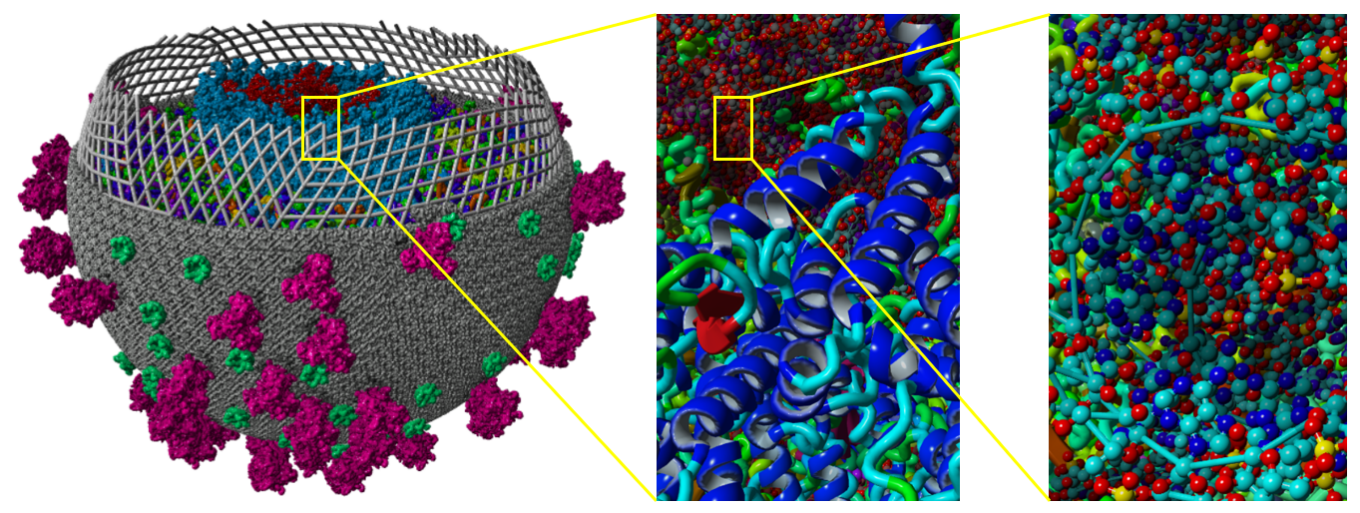
YASARA View (http://www.yasara.org/ (accessed on 15 October 2023)) [16] is used to visualize HIV at a mesoscopic level. The LOD technique is used with the appropriate level of detail. The color scheme is adjusted adaptively.

Color is commonly used in molecular visualization to visually differentiate components. Waldin et al. [62,63] proposed a dynamic multiscale color mapping technique that adaptively adjusts the color scheme based on the current view and scale, ensuring the optimal representation of structural information at any given scale. CellVIEW [64] and YASARA View [16] combine LOD technology to realize the automatic color conversion of complex biomolecules. Figure 4 showcases an application example of color mapping technology in YASARA View. However, color selection in molecular visualization often depends on cultural factors or personal preference. Inconsistent semantic color spaces can reduce the overall interpretability and effectiveness of molecular visualization. Garrison et al. [65] provide color palette samples for industrial and research sectors and propose considerations for developing best practices in color palettes.

In practical applications of molecular visualization, a combination of various rendering techniques is often used to aid in the analysis of complex structures. MegaMol [14] and Molstar [15] integrate advanced lighting algorithms for the efficient rendering of molecular data, with Molstar being capable of online rendering using multiple algorithms.

### 2.3. LOD Technology

In complex molecular visualization scenarios, when the camera is far from the atoms or when the atoms are scaled down to a volume smaller than one pixel, the projection on the screen becomes very small, resulting in limited visually recognizable information. To maintain visual effectiveness while reducing model complexity and speeding up rendering, the technique of Level of Detail (LOD) can be used. LOD involves using simplified models for distant observation or low zoom levels, reducing the number of vertices and faces to improve rendering performance. For close observation or high zoom levels, more detailed models are used to maintain detail and realism.

Guo et al. [66] proposed an LOD representation method for visualizing atomic structures in biology. They used an approximation error metric to evaluate the error of sphere simplification, achieving high-precision rendering and adaptive LOD selection based on the view. Liang et al. [61] utilized LOD techniques to reduce the number of primitives for rendering molecular surfaces, achieving a hierarchical abstraction representation of large molecules and distance-based LOD selection to ensure symmetrical structures have the same representation. Ozvoldik et al. [32] proposed a mesh-based LOD algorithm for LOD rendering of commonly used models such as ball-and-stick models and cartoon models for particularly large biomolecules.

LOD techniques are effective methods for achieving multi-scale visualization [10] and mid-scale visualization [16], especially when dealing with complex datasets or scenes that require smooth transitions and detail switching between different levels. CellVIEW [64] utilizes the Unity3D game engine to interactively visualize large molecular datasets, automatically selecting appropriate colors and detail levels using innovative LOD techniques to achieve seamless visual transitions between different abstraction levels. Goodsell et al. [16] connect the nanometer scale of molecules with the micrometer scale of cells through mid-scale modeling and visualization to simulate the molecular structure of living cells. LOD techniques are used to select appropriate detail levels and keep computational demands within achievable limits, providing sufficient detail to support recognition and understanding, as shown in Figure 4.

Additionally, LOD techniques can also help achieve high frame rate rendering on immersive visualization devices [67]. Goddard et al. [68] used LOD techniques to render complex molecular scenes at high frame rates in virtual reality environments.

### 2.4. Positional Uncertainty Visualization Technology

Biomolecules exhibit dynamic and flexible behavior, which introduces positional uncertainty. Additionally, the process of collecting and processing data introduces uncertainties. These uncertainties can arise from experimental data due to resolution limitations, simulated data generated by simulation algorithms, and visualized data obtained through analysis algorithms prior to visualization. It is crucial to accurately and honestly represent these uncertainties in visualizations.

**Figure 5:**
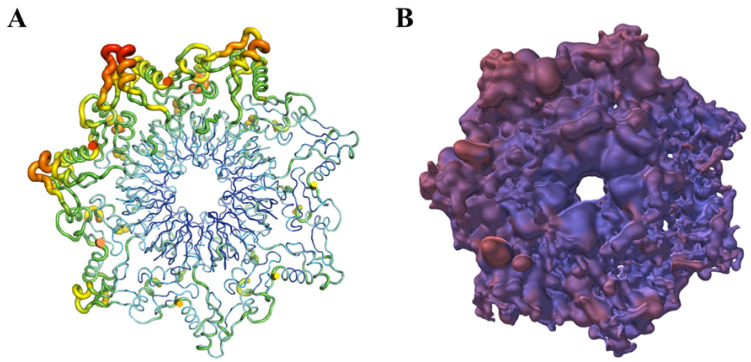
The uncertainty visualization of the bacterial protein (PDB ID: 1M5Q) using PyMol [70] and UnityMol [26]. (**A**) Sausage representation, (**B**) molecular surface.

The dynamics and flexibility of biomolecules can be studied using molecular dynamics simulations. However, these simulations require extensive computing resources and high frame rate rendering algorithms. In current research, processed static structures have been primarily used to visualize the uncertainty in atomic positions, rather than dynamic images or videos. Commonly used representations for depicting this uncertainty in atomic positions in proteins include sausage-like representations, such as the “sausage” view in MolMol [69] and the “putty” representation in PyMOL [70]. These representations allow for the drawing of tubular splines with variable radii, where larger radii indicate greater uncertainty in atomic positions. The color change in the sausage plot can be determined by the B factor, which is a parameter used in X-ray diffraction experiments to describe the position uncertainty caused by thermal vibrations of atoms in a crystal. Higher B-factors in sausage plots generally correspond to larger uncertainties and are represented by cooler tones. These color changes can help observers intuitively understand the uncertainty of atoms at different positions. Additionally, the uncertainty in the molecule can be represented using a molecular surface colored according to the B-factor, as shown in Figure 5.

Sterzik et al. [71,72] mapped uncertainty introduced during data acquisition and processing onto solvent-excluded surface (SES), van der Waals (vdW) surface, and cartoon models using stylized lines. Perception studies were conducted to determine the effectiveness of five line variables (roughness, jitter, grayscale, width, and blur) in distinguishing various uncertainty values in molecular representation models. The results indicated that width and grayscale achieved better results, although grayscale was more sensitive to color changes in the molecular representation models. Roughness and blur only applied to medium-high uncertainty differences, with roughness also requiring less dense lines. Jitter had the least effective outcome. Future work may focus on the combined application of line variables and stylized line generation algorithms, while considering interference with the perception of other characteristics. Schulz et al. [73] represented uncertainty by uniformly distorting the geometric structure of standard molecular representation models in all directions using periodic waveforms, where the uncertainty arose from inconsistencies in secondary structure assignments.

Discrete representation and possibility volume representation are alternative methods for visualizing the uncertainty in atomic positions [74]. Discrete representation treats atomic positions as independent points and uses transparency to express the probability of each position’s existence. Although this approach provides highly detailed and accurate information, the resulting images may be overly complex and difficult to interpret. Possibility volume representation treats atomic positions as a three-dimensional grid in continuous space and colors the grid based on probability density functions. Maack et al. [75] used volume rendering and combined techniques such as transparency and B-factor coloring to visualize the uncertainty in atomic positions, providing a visual analytical framework for protein data with uncertainty. This uncertainty captures variations in atomic positions due to imprecise measurements or multi-model calculations.

### 2.5. Immersive Visualization Technology

Molecular immersive visualization utilizes Virtual Reality (VR) or Augmented Reality (AR) technologies to visualize molecular structures, enhancing the observer’s intuitive perception of complex structures and spatial relationships.

VR technology places users in a completely virtual and immersive simulated environment, isolated from the real environment. Users typically wear specialized VR devices such as head-mounted displays, sensors, and headphones to experience the virtual environment. Kuťák et al. [11] provide a detailed overview of molecular immersive rendering using modern head-mounted platforms and list a range of available tools. Game sensors [76] and force feedback gloves [77] enable hand-controlled molecular virtual reality.

On the other hand, AR technology superimposes virtual elements onto the real world to expand users’ perception of the real world. This can be achieved on mobile devices such as smartphones and tablets, for example, StereoChem [78] and Augment [79].

The development and reduction in the price of computer hardware, especially head-mounted displays, have led to the emergence of numerous molecular immersive visualization technologies and tools. One approach to developing these tools is integrating existing tools into immersive environments. For example, Dimmol is based on UnityMol [80], ChimeraX is based on Chimera [68], and CootVR is based on Coot [81]. To provide a smooth visual experience, molecular immersive visualization requires rendering technologies that support low latency and high frame rates. Stone et al. [82] propose a high-performance rendering method that combines techniques such as ray tracing and occlusion to achieve immersive molecular visualization and avoid network latency. Web-based molecular immersive visualization tools have also been developed to facilitate user access. Examples include ProteinVR [83] and prototype web applications for AR molecular modeling [84].

Fombona-Pascual et al. [85] provide a detailed review of molecular virtual reality and virtual reality laboratories, highlighting the tremendous potential for development in virtual reality laboratories.

## 3. Visualization Tools

Exploring the structure of biomolecules requires the use of several useful tools. This section will introduce three aspects: three-dimensional graphics programming interfaces, offline software or programs, and web-based visualization tools.

### 3.1. 3D Graphics Programming Interface

Visualization is the process of transforming data into visual representations using computer graphics techniques. Computer graphics provides technical support and tools for visualization, and developers often use graphics-related interfaces to write and implement molecular visualization.

OpenGL [86] is a low-level graphics library used for rendering 2D and 3D graphics that is cross-language and cross-platform. It is widely used due to its powerful functionality and mature development compared to other graphics APIs. GLSL [87] is the built-in shading language of OpenGL, used to write shader programs in the graphics rendering pipeline. Molecular visualization software such as VMD [5], PyMol [70], and UCSF Chimera [88] predominantly use OpenGL for visualizing molecular structures. To cater to the graphics rendering needs of mobile devices, game consoles, and embedded platforms, OpenGL ES [89] has been optimized for hardware and resource constraints.

With the rapid development of web applications, there is increasing interest in web-based graphics rendering. WebGL [90] emerged as part of HTML5 technology [91]. It allows for the real-time rendering of 3D graphics in web browsers by embedding the OpenGL ES API into JavaScript. This technology enables developers to create interactive 3D graphics applications in the browser without the need for plugins or additional software. WebGL combines the advantages of OpenGL and JavaScript, facilitating the fast and convenient rendering of high-quality graphics. JSmol [8] and 3Dmol [92] are implemented using WebGL. Additionally, WebGL-based graphics libraries like Three.js [93] have emerged to simplify the implementation of complex 3D scenes and effects.

Vulkan [94] is a next-generation low-level graphics and compute API that offers more control and optimization opportunities compared to OpenGL. It is more low-level and high-performance. Vulkan provides better multithreading support, lower CPU overhead, higher graphics rendering performance, and lower driver overhead. NVIDIA’s Vulkan driver supports the Vulkan RT extension on some GPUs, allowing developers to utilize hardware-accelerated ray tracing technology in the Vulkan API. YASARA [16] uses Vulkan for the assembly and visualization of biomolecular megastructures.

### 3.2. Offline Software or Programs

Molecular visualization techniques and analytical methods are often integrated into software to offer a comprehensive representation of molecule structure and properties. Therefore, the research has primarily focused on developing feature-rich software. Table 2 presents a list of commonly used offline molecular visualization software.

### 3.3. Web-Based Tools

Currently, molecular visualization software is powerful but primarily offline, as it needs to be installed on supported platforms. This limitation hinders real-time sharing among users. On the other hand, WebGL enables developers to create interactive 3D graphics applications directly in the browser. WebGL-based molecular visualization tools offer the convenience of cross-platform usage without the need for plugins or additional software. Table 3 presents some web-based visualization tools.

One notable WebGL-based interactive visualization tool is JSmol [8]. It is a JavaScript implementation of Jmol [95] that eliminates the need for Java installation. Another tool, Abriata [96], combines JSmol and Peer.js to create a visualization page where multiple users can simultaneously view and manipulate 3D molecular structures in their respective browsers. It is worth mentioning that RCSB PDB (https://www.rcsb.org/ (accessed on 15 October 2023)) recommends three molecular viewers: JSmol, NGL, and Molstar [97].

## 4. Future Challenges

Over the past several decades, the field of molecular visualization has matured thanks to advancements in computer hardware and technology. However, it still faces numerous challenges.

The visualization of molecular data has become increasingly complex, encompassing everything from individual molecules to intricate biomolecular landscapes. Additionally, the data extends from static structures to time-dependent molecular dynamics simulation data. This complexity is expected to continue growing, placing greater demands on hardware and technology. To address these demands, more sophisticated systems and integrated approaches are required. Multiscale visualization methods that connect spatial and temporal scales are necessary to gain a deeper understanding of the multidimensional characteristics of biological molecular systems [23]. Furthermore, when visualizing molecular assemblies, Cryo-EM still relies on manual selection and coloring of individual chains. A new approach that is more easily connected to functional annotations is needed [12].

Moreover, the increasing complexity of dynamic simulation data will drive the development of new visualization methods. State-of-the-art hardware that supports the combination of ray tracing and Monte Carlo image denoising techniques can enable interactive path tracing while ensuring interactivity and image quality [50]. Describing the potential uncertainty of molecular graphics and displaying the data sources is also a significant challenge for the future, as it is crucial for accurately acquiring knowledge. Recent research [72] proposes the use of stylized lines to visualize positional uncertainty, however, the generation algorithm for lines and the exploration of the most efficient line styles still require further study.

In the development and presentation of molecular graphics, rendering, coloring, human–computer interfaces, and narrative methods are continuously evolving, resulting in enhanced expressive effects. However, widely used visualization tools still rely on basic aesthetic methods, with most users relying on default rendering and coloring. Therefore, the setting of default values and presets, as well as additional artistic guidance, are essential for creating visually appealing molecular graphics. In the future, molecular visualization will incorporate more creativity and artistic elements to make the presentation of molecular data more aesthetically pleasing and expressive, appealing to a wider audience.

The advancement of hardware devices such as head-mounted displays and VR/AR-related technologies will immerse users in more immersive visualization scenes, enhancing their perception of molecular structures through virtual and interactive human–computer interactions. Stronger support for visualization technology is needed to ensure the display and fluency of high-quality graphics in immersive visualization scenes. Sound engineering can also enhance users’ immersion in virtual scenes. Interestingly, sounds can be used to emphasize events in molecular dynamics simulations. For example, breaking bonds can be represented by a snapping sound in Molecular Zoo [68]. There are also studies on the docking of ligands at protein binding sites. The distance between a user-defined molecule and the binding site is extracted, and a sound is played to notify the user of the distance [98]. Considering how to integrate audio and other sensory modes to improve the accessibility of molecular graphics is an interesting topic for enhancing our understanding of molecules. Furthermore, molecular animation plays an increasingly important role in scientific visualization and scientific communication by conveying non-fictional documentary-type stories [99]. Molecular animation needs to incorporate expertise from various fields, such as animation design, molecular representation, and molecular dynamics.

Molecular visualization also requires collaboration among multiple disciplines. Strengthening the exchange, collaboration, and knowledge sharing among computer graphics, structural biology, educational science, and other fields is currently a focal point, and this collaboration is constantly expanding. For example, incorporating annotations from bioinformatics experts, best practices from perceptual science and science historians, as well as direct user feedback from education assessment experts [12], has become a trend in molecular visualization research. As students are the primary users of molecular visualization tools, their evaluation and feedback are crucial in visualization research. Developing educational use cases, collecting students’ usage experiences, and analyzing learning outcomes can greatly enhance molecular visualization tools and contribute to the advancement of molecular visualization research [11].

## 5. Conclusions

The field of molecular visualization has made significant progress in recent years. This article provides a review of its historical development, related techniques, tools, and future challenges.

We have observed the evolution of molecular visualization, from physical models to highly accurate computer-generated images. These images have become powerful tools for scientific research, drug design, and science communication. Various advanced computer graphics techniques, virtual reality technology, and deep learning algorithms have further improved the quality and interactivity of molecular visualization. Additionally, there are numerous excellent visualization tools available for researchers, offering a wide range of options for both basic scientific research and applied research. This allows researchers to find tools that suit their needs.

Looking ahead, molecular visualization, as an interdisciplinary field, plays an irreplaceable role in scientific research and applications. With the continuous improvement of computational capabilities, we can expect the emergence of more precise and faster visualization methods in the near future.

## Figures and Tables

**Table 1:** List of review papers on molecular visualization.

Paper	Graphics	Biology	Chemistry	Education	Drug Design	Aesthetics
Johnson et al. [18]	✓	✓				
Alharbi et al. [20]	✓	✓				
Kozlíková et al. [17]	✓	✓				
Yuan et al. [21]	✓	✓	✓		✓	
Jenkinson [22]	✓	✓		✓		
Olson [3]	✓	✓				
Schatz et al. [23]	✓	✓				
Martinez et al. [19]	✓	✓				
Miao et al. [10]	✓	✓				
Martinez et al. [24]	✓	✓				
Shen [25]	✓	✓	✓	✓		
Garrison et al. [12]	✓	✓				✓

**Table 2:** Offline software or programs.

Software or Program	Description	Link
VMD	Visualization system for visualizing, analyzing, and animating large biomolecular data	https://www.ks.uiuc.edu/Research/vmd/ (accessed on 15 October 2023)
PyMol	Molecular visualization system based on open source	https://pymol.org/2/ (accessed on 15 October 2023)
UCSF Chimera	Interactive visualization and analysis of molecular structures and related data	https://www.cgl.ucsf.edu/chimera/ (accessed on 15 October 2023)
MegaMol	Visualization framework with advanced lighting algorithms	https://megamol.org/ (accessed on 15 October 2023)
UnityMol	Molecular viewer created using the Unity3D game engine	http://www.baaden.ibpc.fr/umol/ (accessed on 15 October 2023)
YASARA	High-performance visualization of ultra-large molecular data, supports virtual reality, offers a free version called YASARA View	http://www.yasara.org/ (accessed on 15 October 2023)
BALLView	Molecular modeling and visualization program for BALL	https://ball-project.org/ballview/ (accessed on 15 October 2023)
cellVIEW	Visualization and multiscale rendering tool for large biomolecular datasets	https://www.cg.tuwien.ac.at/page/cellview/ (accessed on 15 October 2023)
CAVER Analyst	Calculation, analysis, and real-time visualization of tunnels in static and dynamic protein structures	https://www.caver.cz/index.php?sid=100 (accessed on 15 October 2023)
QuteMol	Open source, interactive, high-quality molecular visualization system	https://qutemol.sourceforge.net/ (accessed on 15 October 2023)
ProteinShader	Generate illustrative renderings of proteins	https://proteinshader.sourceforge.net/index.php (accessed on 15 October 2023)
MolecularNode	Quick import and visualisation of structural biology data inside of Blender	https://github.com/BradyAJohnston/MolecularNodes (accessed on 15 October 2023)
mMaya	Molecular modeling, animation, and simulation plugin based on Maya	https://clarafi.com/tools/mmaya/ (accessed on 15 October 2023)
MolSoft ICM-Pro	High-quality protein visualization, modeling, and structure analysis tools	https://molsoft.com/icm_pro.html (accessed on 15 October 2023)
ePMV	Open-source uPy plugin, embedded Python molecular viewer	http://epmv.scripps.edu/ (accessed on 15 October 2023)
Pyrite	Blender plugin that can import atomic motion captured from molecular dynamics simulations	https://durrantlab.pitt.edu/pyrite/ (accessed on 15 October 2023)

**Table 3:** Web-based tools.

Tool	Description	Link
NGL Viewer	Collection of molecular graphics tools for visualizing various representations of molecular structures	http://nglviewer.org/ (accessed on 15 October 2023)
JSmol	JavaScript implementation of Jmol, a molecule viewer	https://jmol.sourceforge.net/ (accessed on 15 October 2023)
3Dmol	Molecular visualization JavaScript library that provides a fully-featured API	http://3dmol.csb.pitt.edu/ (accessed on 15 October 2023)
EzMol	Quickly generates high-resolution images of proteins	http://www.sbg.bio.ic.ac.uk/ezmol/ (accessed on 15 October 2023)
LiteMol	Rapid visualization of macromolecular structures	https://litemol.org/ (accessed on 15 October 2023)
iCn3D	3D viewer for macromolecular structures and chemical substances	https://structure.ncbi.nlm.nih.gov/Structure/icn3d/ (accessed on 15 October 2023)
ChemDoodle	Small open-source JavaScript library for fast, professional, and online molecular structure drawing	http://web.chemdoodle.com/ (accessed on 15 October 2023)
PDBms	Reads atomic coordinates from PDB files and enables visualization and manipulation of each atom	https://www.biogem.org/tool/pdbms/ (accessed on 15 October 2023)
Molstar	Open-source toolkit for visualization and analysis of macromolecular data	https://molstar.org/ (accessed on 15 October 2023)
Illustrate	Non-photorealistic molecular illustration with cartoon colors, outlines, and soft ambient shading	https://ccsb.scripps.edu/illustrate/ (accessed on 15 October 2023)
Speck	Feature-rich molecular renderer	http://wwwtyro.github.io/speck/ (accessed on 15 October 2023)
PV	Protein structure viewer	https://biasmv.github.io/pv/ (accessed on 15 October 2023)
MolView	2D and 3D molecular structure editor and viewer with integration of other molecular viewers	https://molview.org/ (accessed on 15 October 2023)
Miew	Advanced visualization and manipulation of molecular structures	https://miew.app/ (accessed on 15 October 2023)
iMolecule	Python-based molecule viewer	https://github.com/patrickfuller/imolecule (accessed on 15 October 2023)
GLmol	3D molecule viewer	http://webglmol.osdn.jp/ (accessed on 15 October 2023)
Molvwr	Online molecule viewer made with Babylon.js	https://github.com/gleborgne/molvwr (accessed on 15 October 2023)
BioWeb3D	Visualization of large datasets	https://github.com/jbogp/bioWeb3D (accessed on 15 October 2023)
CH5M3D	Drawing and editing of small molecule 3D structures	https://ch5m3d.sourceforge.net/ (accessed on 15 October 2023)
concurrent-jsmol-visualization	Multiple users interactively view and manipulate 3D molecular structures simultaneously through their respective browsers	http://lucianoabriata.altervista.org/jsinscience/concurrent-jsmol/concurrent-jsmol-visualization.html (accessed on 15 October 2023)

## Data Availability

Not applicable.

## Abbreviations

CPU	Central Processing Unit
GPU	Graphics Processing Unit
vdW	van der Waals
SAS	Solvent Accessible Surface
SES	Solvent Excluded Surface
AO	Ambient Occlusion
LOD	Level of Details
VR	Virtual Reality
AR	Augmented Reality
OpenGL	Open Graphics Library
OpenGL ES	OpenGL for Embedded Systems
WebGL	Web Graphics Library
API	Application Programming Interface
